# The effects of emotion regulation and coping skills on mental health in refugee children: A cross-sectional study

**DOI:** 10.1097/MD.0000000000044856

**Published:** 2025-09-26

**Authors:** Sümeyye Belhan Çelik, Esma Özkan

**Affiliations:** a Department of Occupational Therapy, Faculty of Hamidiye Health Sciences, University of Health Sciences, İstanbul, Türkiye; b Department of Occupational Therapy, Faculty of Gülhane Health Sciences, University of Health Sciences, Ankara, Türkiye.

**Keywords:** coping skills, emotion regulation, mental health, refugee child

## Abstract

Refugee populations worldwide are exposed to violence and trauma, leading to severe mental health challenges. Many refugee children experience multiple traumatic events and human rights violations, which increase their risk for emotional and behavioral difficulties. Emotion regulation and coping skills play a critical role in maintaining and improving mental health for children facing such risks. Although there is growing literature on the mental health of refugee children, important factors like emotion regulation and coping have not been sufficiently addressed. This study examines the effects of emotion regulation and coping on mental health among 120 refugee children (mean age 10.91 ± 1.61). Participants completed the Demographic Information Form, the Emotion Regulation Checklist, the Coping Scale for Children and Youth, and the Strengths and Difficulties Questionnaire. Results revealed a significant medium to strong relationship between emotion regulation, coping skills, and mental health outcomes (*P* < .001). Regression analysis indicated that emotion regulation and coping skills explained 83.3% of the total difficulties score. These findings demonstrate that refugee children with stronger emotion regulation and coping abilities report fewer problems in attention, emotional, behavioral, peer, and social areas. This highlights the importance of interventions that build these skills to reduce the negative impacts of trauma. Health professionals should prioritize programs that enhance emotion regulation and coping to support the psychological well-being and adaptation of refugee children, who are among the most vulnerable to the consequences of war trauma.

## 1. Introduction

Migration is characterized by forced and often sudden evacuations and may involve risks arising from geography (e.g., crossing deserts, mountains, rivers), encounters with wild animals, increased conflict, and lack of essential resources. This phase may also involve prolonged stays in refugee camps or urban centers in countries of first asylum, where discrimination and inadequate access to food, water, safety, and education are common. In addition, refugees face risks such as thirst, hunger, extreme weather conditions, and may witness the deaths of their comrades.^[1,2]^

Pre-migration and migration experiences can lead to a variety of negative outcomes. Children from refugee families, for instance, could be afraid of authority figures and turn to solitude as a result of their interactions with informants and undercover police. Children who are involved in conflict may sustain physical harm. Early life malnutrition can cause irreversible mental impairment, and school disruption can harm students’ academic performance.^[3]^ Cultural grieving and acculturation stress are 2 additionalpost-migration stressors.^[1]^ Children of refugees frequently assume adult tasks such as supporting their families, translating for their parents, or starting employment because they frequently pick up the host language faster than their parents.^[4]^ Furthermore, a lot of refugee kids deal with their constant separation from friends and family as well as the unpredictability of losing family members whose whereabouts are unknown.^[3]^

Over half of the world’s 25.9 million refugees in 2018 were under the age of 18, according to the United Nations High Commissioner for Refugees. Refugee populations around the world are exposed to violence and trauma. Research has demonstrated that a significant number of traumatic occurrences are experienced by refugees who become victims of human rights violations. It has been discovered that trauma exposure causes serious mental health issues in all populations.^[5]^ Children of refugees may encounter what is known as the “cumulative stress of displacement,” which is the result of combining early life stresses with extreme and traumatic experiences of displacement. As a result, these children are at greater risk of psychological distress than non-refugee children.^[6]^ Emotion regulation and coping strategies for children living with these risks play an important role in maintaining and promoting mental health.^[7–9]^ The function of emotion regulation skills is to ensure the satisfaction of needs, facilitate goal achievement, and assist self-actualization.^[7]^ Children’s ability to control their emotions and behaviors is crucial for their social skills, acceptability by their peers, academic performance, and adjustment to everyday life.^[8]^ The ability to control one’s emotions evolves with age and experience. The deliberate response to stress shapes coping mechanisms. They control how stress and health are related.^[10]^ According to Lazarus, coping methods are employed to control behavior, emotion, and thought processes in stressful situations.^[11]^ Three primary headings were used by Eisenberg and colleagues to analyze children’s coping mechanisms: emotion-focused coping, problem-focused coping, and controlling behavior linked to emotions. The goal of problem-focused methods is to alter the stressor or the surrounding circumstances. By controlling the child’s perception of stress, emotion-focused strategies seek to help them adjust to the stressor. Rather of altering the stressful environment or controlling stressful events, emotion-related behavior regulation seeks to control behaviors that might elicit unpleasant emotions.^[12]^ Since coping and emotion regulation share the same framework, it is crucial to examine them jointly in research on children’s mental health.

The literature on the mental health of refugee children includes several studies,^[13,14]^ but it is evident that when addressing life events that may affect mental health in these studies, crucial factors like emotion regulation and coping are not addressed and need to be looked into. Thus, this study aims to examine the effects of emotion regulation and coping on mental health in refugee children. This research is anticipated to provide insights into the topics of emotion regulation and coping strategies for practitioners in the field of mental health among refugee children.

The H₁ hypotheses of our study are as follows:

1. Refugee children with higher levels of emotion regulation will exhibit fewer emotional, behavioral, and social difficulties.
2. Refugee children with higher levels of coping skills will exhibit fewer emotional, behavioral, and social difficulties.
3. Emotion regulation and coping skills together will significantly predict and explain a substantial portion of the variance in refugee children’s mental health outcomes.

## 2. Methods

### 2.1. Study design

Our study was designed as a cross-sectional design. The study protocol was approved by the University of Health Sciences Hamidiye noninvasive Investigation Ethics Committee (23/643-01.12.2023). An informed consent form was prepared to notify and inform the parents prior to administering the surveys to these children. The parents of the refugee children were informed about our study’s purpose and methods and informed consent form was signed by the parents on enrollment of their children because they were under the age of 16. The ability to leave the study at any time and without explanation was made clear to the participants. Data were gathered in person. One of the basic sampling techniques, the snowball/chain sampling approach, was used to recruit the participants.

### 2.2. Participants

**Figure 1.**
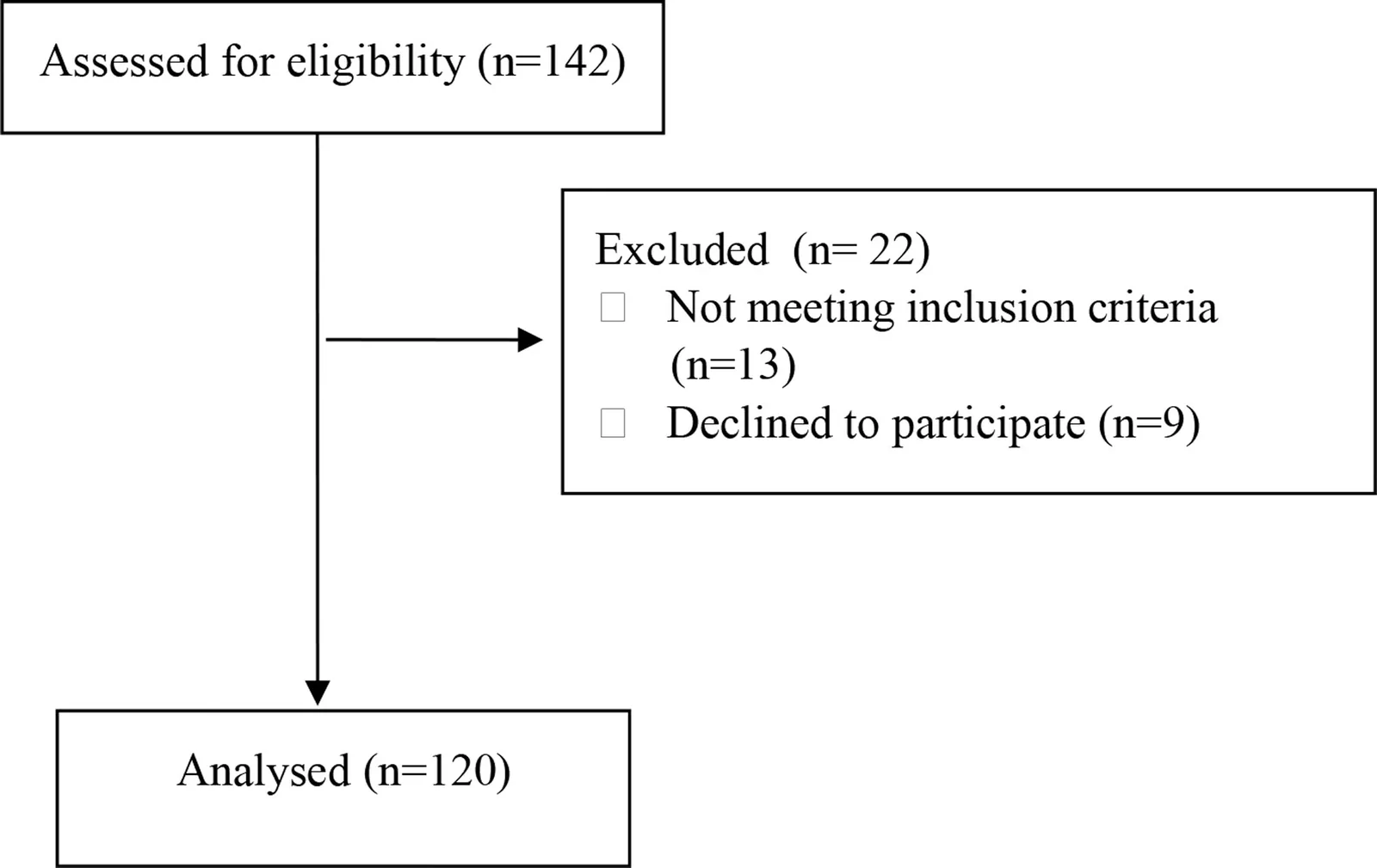
Flow diagram of participant recruitment and analysis. Of 142 participants assessed for eligibility, 22 were excluded (13 did not meet inclusion criteria; 9 declined to participate). A total of 120 participants were included in the analysis.

The G*Power (version 3.1.9.2; Heinrich-Heine-University of Düsseldorf, Düsseldorf, Germany) package program was used to determine the sample size that we used in our investigation. A minimum of 120 people was determined, based on an effect size of 0.3, a Type I error rate of 0.05, and a power of 0.90.^[15]^ The 120 refugee children who were living in the community and in Istanbul and Ankara made up the study’s population. Being between the ages of 7 and 13; having immigrated to Turkey as a result of war in their home country; being literate in Turkish; and the child and their family voluntarily agreeing to take part in the study were the inclusion criteria for the research. Children enrolled in supplemental schooling or therapy programs, as well as those with any neurological, developmental, or learning disabilities, were excluded from the study. For our investigation, 142 children were interviewed and the data collection process was terminated when the targeted number of participants was obtained through g power analysis (Fig. 1).

### 2.3. Outcomes

The Demographic Information Form, the Emotion Regulation Checklist (ERC), the Coping Scale for Children and Youth (CSCY), and the Strengths and Difficulties Questionnaire (SDQ) were administered to children who consented to participate in the study.

#### 2.3.1. Demographic information form

Prepared by the researcher. It includes questions to determine the sociodemographic characteristics of the children (age, gender, education level, employment status of parents, etc).

#### 2.3.2. The ERC

ERC was developed by Shields and Cicchetti in 1997.^[16]^ The Turkish adaptation of the ERC for the 6 to 13 age group was made by Kapçi et al.^[17]^ It evaluates emotional reactions and the regulation and expression of emotions according to the conditions of the environment. Using a Likert-type scale with a range of 1 to 4, the mother, father, teacher, or another adult who is familiar with the child assesses how frequently they see the behaviors mentioned on the scale in the child. *Higher scores signify greater emotional self-regulation capacity.* High reliability was found, with a Cronbach alpha value of .94 for the lability/negativity subscale and .88 for the overall scale^[17]^

#### 2.3.3. The CSCY

The CSCY is based primarily on the theoretical approach and studies of Lazarus and Folkman on coping for adults.^[11]^ A 29-item scale was developed by Brodzinsky et al to determine the coping styles of children and adolescents.^[18]^ The correlation values obtained for the test–retest reliability of the CSCY ranged from .32 to .67. It has been shown that the CSCY is a valid and reliable measurement tool to be used in determining the coping styles of adolescents in our country. The items in the scale are answered on a 4-point Likert-type scale as never (1), sometimes (2), often (3), and always (4). High scores indicate that individuals can better cope with a stressful or traumatic situation.^[19]^

#### 2.3.4. The SDQ

SDQ was created by Robert Goodman in 1997, is another measure used to screen for mental health issues in children and adolescents.^[20]^ This questionnaire has a parent form and teacher form for ages 4 to 16, and an adolescent form filled out by the adolescent himself/herself for ages 11 to 16. In our study, the parent form was used. There are 25 questions in the SDQ, a mix of positive and negative questions that probe behavioral characteristics. These questions are grouped under 5 subheadings, namely emotional difficulties, conduct difficulties, hyperactivity/inattention, peer relationship difficulties, and social behavior difficulties. Each heading is evaluated individually, and the sum of the first 4 headings gives the “total difficulty score.” A lower score indicates better mental health.^[21]^

### 2.4. Statistical analyses

All data were presented as mean ± standard deviation (SD) or relevant statistical measures, and statistical significance was set at *P *< .05. As descriptive statistics, mean and standard deviation (mean ± SD) were used for continuous data, and frequency and percentage were used for categorical data. The conformity of continuous data to normal distribution was checked with the Kolmogorov–Smirnov test. The relationships between variables were evaluated with Spearman correlation analysis and the following cutoff points were used for the interpretation of correlation coefficients; 0.00 to 0.19 very weak, 0.20 to 0.39 weak, 0.40 to 0.69 moderate, 0.70 to 0.89 high, and 0.90 to 1.00 very high relationship.^[22]^ The estimation model was obtained using the multiple linear regression analysis and the stepwise variable selection method. Normal distribution was examined with the Kolmogorov–Smirnov test, and the linear relationship was examined with the scatter plot. The adequacy of the model was assessed using the Breusch–Pagan test for homogeneity of variance (homoscedasticity), the Durbin–Watson test for correlation between errors (autocorrelation) and the variance inflation factor. Effective and distant observations were examined using Cook distance and covariance ratio, and extremely outlier observations were examined using standardized residual plots. The suitability of the errors for normal distribution was decided using the Kolmogorov–Smirnov test on standardized residuals.^[22]^ IBM SPSS Statistics for Windows, Version 23.0 (IBM Corp., Armonk, NY, USA) was used to evaluate the data.

## 3. Results

This study analyzed a sample of 120 participants and found that the age of participants ranged from 7 to 13 years, with an average age of 10.91 ± 1.61 years. Table 1 presents additional sociodemographic variables.

**Table 1:** Demographic characteristics.

N = 120		n	%
Gender	Male	58	48.3%
	Female	62	51.7%
Age (mean ± SD) 10.91 ± 1.61	8	8	6.7%
	9	21	17.5%
	10	22	18.3%
	11	19	15.8%
	12	23	19.2%
	13	27	22.5%
Country of origin	Syria	120	100%
Economic status	Expence > Income	16	13.3%
	Expence = Income	80	66.7%
	Expence < Income	24	20.0%
Parental highest education level	No formal education	18	15.0%
	Primary education	52	43.33%
	Secondary education	36	30.0%
	Higher education	14	11.67%
Parental employment status	Both parents unemployed	32	26.67%
	At least 1 parent employed	62	51.67%
	Both parents employed	26	21.67%

### 3.1. Correlation analysis

The relationship between mental health and emotion regulation and coping skills was evaluated using the Spearman correlation coefficient (Table 2). A very strong negative relationship (rho= 0.814, 0.723, and 0.753, respectively) was found between the strengths–difficulties total and its sub-dimensions, emotional problems, behavioral problems, and emotion regulation (*P* < .001). A statistically significant negative relationship of moderate strength (rho; 0.679, 0.654, and 0.609, respectively) was found between the attention deficit, peer problems, and social behavior sub-dimensions of the strengths–difficulties scale and emotion regulation (*P* < .001). A negative and very strong (rho; 0.865, 0.783, 0.800, 0.763, and 0.827) statistically significant relationship was found between coping skills and the total SDQ, attention difficulties, emotional difficulties, conduct difficulties, and social behavior difficulties (SBD) sub-dimensions, respectively. A negative and moderately strong (0.684) statistically significant relationship was found between peer relationship difficulties sub-dimension and coping skills (*P* < .001) (Table 2). In addition, a positive and strong relationship was found between the participants’ emotion regulation and coping skills (*P* < .001, rho:0.740).

**Table 2:** The results of correlation analysis.

N = 120		SDQ TOTAL	Attention difficulties (AD)	Emotional difficulties (ED)	Conduct difficulties (CD)	Peer relationship difficulties (PRD)	Prosocial behaviour (PB)
ERC	Rho	−0.814*	−0.679*	−0.723*	−0.753*	−0.654*	−0.609*
	*P*	**<.001**	**<.001**	**<.001**	**<.001**	**<.001**	**<.001**
CSCY	Rho	−0.865*	−0.783*	−0.800*	−0.763*	−0.684*	−0.827*
	*P*	**<.001**	**<.001**	**<.001**	**<.001**	**<.001**	**<.001**

CSCY = Coping Scale for Children and Youth, ERC = Emotion Regulation Checklist, SDQ = Strengths and Difficulties Questionnaire. Bold values indicate statistically significant correlation (*P* < .05).

* Rho: Spearman test statistical value, statistical significance (*P* < .05).

### 3.2. The results of regression analysis

The regression model formed by the variables affecting the SDQ Total Score was found to be significant (*P* < .001). The independent variables in the model explain 83.3% of the change in the SDQ Total Score. According to the standardized regression coefficients (Beta), the Coping variable (-0.622) made the greatest contribution to the model. A 1-unit increase in the Coping variable caused a 0.622 standard deviation decrease in the SDQ Total Score, and a 1-unit increase in the emotion regulation variable caused a 0.353 standard deviation decrease in the SDQ Total Score (Model; SDQ Total Score = 53.402 - 0.318 × coping - 0.164 × emotion regulation) (Table 3).

**Table 3:** The results of multivariate regression analysis for SDQ.

		b	SE (b)	*β*	*t*	*P*	VIF	*R* ^2^	*F*
SDQ total									
(Constant)		53.402	1.209		44.174	.000		0.833	*F*(2117) = 296.883 *P* < .001
CSCY		−.318	.028	−.622	−11.209	.000	2.188		
ERC		−.164	.026	−.353	−6.368	.000	2.188		
SDQ inattention									
(Constant)	13.036		.491		27.458	.000		0.649	*F*(3116) = 110.914 *P* < .001
CSCY	−.0851		.011	−.634	−7.886	.000	2.731		
ERC	−.027		.010	−.219	−2.731	.003	2.211		
SDQ emotional difficulties									
(Constant)	13.194		.445		29.677	.000		0.682	*F*(2117) = 125.554 *P* < .001
CSCY	−.082		.010	−.610	−7.909	.000	2.188		
ERC	−.033		.009	−.266	−3.455	.001	2.188		
SDQ conduct difficulties									
(Constant)	13.933		.542		25.709	.000		0.654	*F*(2117) = 110.503 *P* < .001
CSCY	−.078		.013	−.491	−6.108	.000	2.188		
ERC	−.054		.012	−.375	−4.662	.000	2.188		
SDQ peer relationship difficulties									
(Constant)	13.228		.672		19.687	.000		0.520	*F*(2117) = 62.737 *P* < .001
CSCY	−.070		.016	−.424	−4.291	.000	2.352		
ERC	−.052		.015	−.345	−3.493	.001	2.352		
SDQ prosocial behaviour									
(Constant)	11.940		.388		30.781	.000		0.783	*F*(1109) = 394.351 *P* < .001
ERC	−.128		.006	−.885	−19.858	.000	1.000		

b = unstandardized coefficients, CSCY = Coping Scale for Children and Youth, ERC = Emotion Regulation Checklist, *F* = *F* value, *p* = *P* value, *R*^2.^ = coefficient of determination, SDQ = Strengths and Difficulties Questionnaire, SE(b) = Standard Error of the unstandardized coefficient, *t* = *t* value, VIF = Variance Inflation Factor, *β* = standardized beta coefficients.

The regression model explaining the change in the SDQ attention difficulties (AD) variable was found to be significant (*F*(3.116) = 110.914, *P* < .001). The contribution of the ERC and CSCY variables to the model showing the effect on the SDQ-AD score was found to be statistically significant (respectively; *P* < .001, *P* = .007). The independent variables in the model explain 64.9% of the change in the SDQ-AD score. According to the standardized regression coefficients (Beta), the CSCY variable (-0.634) made the greatest contribution to the model (Table 3).

A 1-unit increase in the CSCY variable caused a 0.634 standard deviation decrease in the SDQ-AD score and a 1-unit increase in the ERC variable caused a 0.219 standard deviation decrease in the SDQ score (Model; SDQ-AD = 13.036 - 0.085 × CSCY - 0.027 × emotion regulation). The regression model explaining the change in the SDQ emotional difficulties (ED) variable was found to be significant (*F*(2.117) = 125.554, *P* < .001). The independent variables in the model explained 68.2% of the change in the ED score. According to the standardized regression coefficients (Beta), the CSCY variable (-0.610) made the greatest contribution to the model. A 1-unit increase in the CSCY variable caused a 0.610 standard deviation decrease in the SDQ-ED score, and a 1-unit increase in the ERC variable caused a 0.266 standard deviation decrease in the SDQ-ED score (Model; SDQ-ED = 13.194 - 0.082 × CSCY - 0.033 × ERC) (Table 3).

The regression model explaining the change in the SDQ conduct difficulties (CD) variable was found to be significant (*F*(2.117) = 110.503, *P* < .001). The independent variables in the model explained 65.4% of the change in the SDQ-ED score. According to the standardized regression coefficients (Beta), the CSCY variable (-0.491) made the greatest contribution to the model. A 1-unit increase in the CSCY variable caused a 0.491 standard deviation decrease in the SDQ-CD score and a 1-unit increase in the ERC variable caused a 0.375 standard deviation decrease in the SDQ-CD score (Model; SDQ-CD = 13.933 - 0.078 × CSCY - 0.054 × ERC) (Table 3).

The regression model explaining the change in the SDQ peer relationship difficulties (SDQ-PRD) variable was found to be significant (*F*(2.117) = 62.737, *P* < .001). The independent variables in the model explained 52.0% of the change in the PRD score. According to the standardized regression coefficients (Beta), the CSCY variable (-0.424) made the greatest contribution to the model. A 1-unit increase in the coping variable caused a 0.424 standard deviation decrease in the SDQ-PRD score and a 1-unit increase in the ERC variable caused a 0.345 standard deviation decrease in the SDQ-PRD score (Model; SDQ-PRD = 13.228 - 0.070 × CSCY - 0.052 × ERC) (Table 3).

The regression model explaining the change in the SDQ-SBD variable was found to be significant (*F*(1.109) = 394.351, *P* < .001). The independent variables in the model explain 78.3% of the change in the SBD score. According to the standardized regression coefficients (Beta), a 1-unit increase in the CSCY variable caused a 0.885 standard deviation decrease in the SDQ-SBD score (Model; SDQ-SBD = 11.940 - 0.128 × CSCY) (Table 3).

## 4. Discussion

The findings of this study offer insights into the variables that might influence the mental health of refugee children. Our research has demonstrated a correlation between the mental well-being of refugee children and their ability to regulate emotions and cope with challenges. Furthermore, this research has shown that factors such as emotion control, and coping skills significantly impact several aspects of mental health, including behavioral difficulties, attention deficit and hyperactivity, emotional problems, peer problems, and social behavior problems. Refugee children who can effectively manage and articulate their emotions, adapt their emotional responses to different environmental circumstances, and employ cognitive and behavioral strategies to solve problems are more likely to exhibit improved emotional, social, and behavioral outcomes in terms of their psychosocial functioning.^[23–25]^

When the sociodemographic findings in our study are evaluated, it is thought that the sample group in our study reflects the diversity in this population in terms of demographic and socioeconomic aspects quite well. Sociodemographic factors like income, education level, and race/ethnicity are linked to health outcomes.^[26]^ Accordingly, in psychological research, providing demographic data without a clear context or sufficient description may cause fundamental findings to be interpreted incorrectly.^[27]^ Comprehending the heterogeneity of sociodemographic attributes among marginalized populations, like immigrants, is crucial in comprehending the factors that influence social health and mitigating health disparities.

The literature does not contain any research that specifically looks at how coping strategies and emotion regulation affect the mental health of refugees. Research on mental health issues related to trauma are more prevalent in the literature. Studies on refugees’ mental health have highlighted the significance of emotion control and coping mechanisms.^[24,28–30]^ According to the Ryan et al study, refugees who are able to effectively regulate their emotions are better able to manage stress and achieve improved mental health outcomes.^[31]^ Our research indicates a connection between coping mechanisms, emotion control, and mental health. These results show that children with high skills in regulating emotions and using coping strategies are more successful in reducing the difficulties they experience in attention, emotional, behavioral, peer relations, and social areas, and that these children have lower (better mental health) strengths and difficulties questionnaire scores.

Research shows that issues with emotion regulation have a major impact on psychological symptoms and general quality of life.^[32,33]^ Therefore, enhancing the psychological well-being and general quality of life of children and young people can be greatly aided by a thorough investigation of the environmental circumstances that impair their ability to regulate their emotions and cope. Yiğit et al study highlights several aspects that impair the emotion control skills of refugee children. For instance, students may find it challenging to control their emotions due to language barriers, which may limit their capacity to communicate their feelings and comprehend those of others. Furthermore, students’ capacity to control their emotions and cope with academic stress may be impacted by disruptions in their schooling and variations in their preparedness for college. Turkish students highlighted the language barrier, peer relationships, teacher attitudes, aggression, separation, exclusion, cultural differences, and differences in academic readiness levels when asked about the factors affecting Syrian students’ adjustment to school.^[34]^

The hardships of war, violence, separation, and loss cause refugees to undergo severe trauma. The emotional control skills of migrants are weakened by these interpersonal traumas, making them more susceptible to mental health issues. SDQ was used in the study by Karadag and Ogutlu to evaluate the mental health status of Turkish teenagers (n = 70) and Syrian refugees (n = 70). When the findings were analyzed, the SDQ scores in the Syrian refugee group were significantly higher. Poorer mental health is indicated by this outcome.^[35]^ Many challenges that arise in post-migration life, including financial hardships, housing issues, social isolation, and language limitations, have a detrimental impact on refugees’ mental health and exacerbate symptoms of post-traumatic stress disorder (PTSD), despair, and anxiety. Since forced migration is recognized as one of the most traumatic experiences, refugees may be more susceptible to the development of psychopathologies, particularly those involving issues with emotion regulation.^[36]^ Research has demonstrated that people with PTSD have a decreased ability to control their emotions, suggesting that emotion dysregulation is a crucial mechanism underpinning the connection between experiences related to being a refugee and psychological disorders.^[37–39]^ Since children and adolescents are the most vulnerable demographics, war crimes are a significant risk factor for a variety of psychosocial adjustment issues, such as PTSD and emotion dysregulation.^[24]^ PTSD and emotion control are intimately linked to coping mechanisms. Scholars have observed a connection between children’s psychosocial adjustment and the coping mechanisms employed by refugees during stressful situations.^[40]^ The ability to assess, control, feel, express, and develop one’s emotions in a way that promotes healthy functioning is known as emotion regulation.^[41]^ There is evidence that emotion dysregulation in trauma survivors leads to depression, despite the fact that the majority of research on emotion regulation in trauma survivors has been on PTSD.^[42,43]^ This demonstrates how people with PTSD struggle to regulate their emotions, which has a detrimental impact on their overall mental health. According to our research, children who are Syrian refugees and are between the ages of 6 and 13 and who possess stronger coping mechanisms and emotional regulation are more likely to have better mental health. Emotional dysregulation can limit refugees’ capacity to cope with these disorders, making it difficult for them to continue their functionality in daily life. Therefore, it is critical to develop emotional control abilities in order to enhance refugees’ mental health. In order to help refugees regain greater emotional balance and lessen the psychological impacts of their traumatic experiences, psychosocial therapies might be very helpful. In this context, health professionals and social workers, such as occupational therapists and psychotherapists who work with refugees, can implement programs to improve emotional regulation strategies, which can be effective in increasing the general well-being of refugee children.

The results of our study showed that the participants’ ability to control their emotions and their coping mechanisms were strongly positively correlated. Intense emotions during the trauma caused the person to lose control, which may have a negative impact on their coping processes. This was highlighted in a study looking at the association between PTSD, depression symptoms, and emotion regulation skills. Parallel to our research findings, this study suggested that people with emotion regulation issues may experience a negative influence on their coping strategies’ ability to operate effectively, which could lead to an increase in the severity of mental health issues.^[44]^

Our study found a strong negative relationship between coping skills and mental health. While there is conflicting information about the potential benefits of coping techniques for refugees from post-displacement stressors on their mental health, we think that having effective coping mechanisms is crucial for lowering the severity of mental health issues. Similar to our findings, a study involving 4000 refugees relocated in Sweden from Syria showed that improved coping abilities are associated with improved mental health. According to the study, coping mechanisms can somewhat reduce the detrimental impacts on refugees’ mental health that they encounter during the resettlement process. The study by Kirmayer et al emphasizes how coping mechanisms affect refugees’ mental health. The study indicated that these strategies helped refugees obtain employment and adjust to their new lifestyles, which in turn made them feel less depressed and anxious. According to the study, by using these strategies, refugees were able to obtain employment and adjust to their new lifestyles, which in turn made them feel less depressed and anxious.^[28]^ Our findings are supported by this finding, which demonstrates the significance of coping skills in the process of maintaining and improving refugees’ mental health. Refugee children in our study who scored highly on coping skills also scored higher on mental health. Obviously, when examining mental health problems, we believe that the refugee population should be examined multifactorially in order to fully understand the profound effects of forced migration on children and the strategies to cope with these effects. As an instance, children’s coping strategies and adaption processes are adversely affected by the cultural norms of the host country and the cultural peculiarities of the destination country.^[45]^ In refugee studies, a variety of elements, including religious systems, spirituality, and social support networks like family and friends, are frequently seen as crucial coping strategies. The existence and quality of these networks can positively affect a person’s ability to cope and, as a result, their mental health.^[30]^ Another study has stated that the factors that have a significant impact on the coping skills of Syrian children are the language barrier, peer relations, and teacher attitudes and marginalization.^[46]^ In this context, it is necessary to design school- and community-based interventions, psychosocial support services, and special education programs for children in accordance with cultural norms. Furthermore, it is imperative to address mental health development through comprehensive, holistic, and broader measures including family education and the improvement of refugee protection laws.^[47]^

There are some limitations in our study. It is important to note that our study has a cross-sectional design, which means that we cannot determine the temporal impacts or causation potential. Longitudinal studies are necessary to have a more comprehensive understanding of the long-term impact of many influences on the mental well-being of refugee children. Another limitation of our study is that it included only refugees living in Ankara and Istanbul, Turkey's 2 largest cities, among the participants residing in Turkey. While our study encompassed individuals from diverse demographic backgrounds, the presence of refugee children living in Istanbul could restrict the applicability of our findings.

## 5. Conclusion

Our study establishes a correlation between the factors examined and mental health. Our results show that refugee children with high emotional regulation and coping strategies are more successful in reducing the difficulties they experience in attention, emotional, behavioral, peer relations, and social areas, and that these children’s emotional regulation and coping skills have an impact on their mental health. Therefore, reducing the effects of the traumatic experiences experienced by refugee children, who are the most vulnerable group to war trauma, and helping them adapt to their new lives through the development of emotional regulation and coping skills can play a critical role in improving their mental health. We believe that in order to improve the mental health of refugees, it will be crucial to take these skills into account in future intervention research.

## Acknowledgments

The authors sincerely thank all of the participants who participated in this study.

## Author contributions

**Conceptualization:** Sümeyye Belhan Çelik, Esma Özkan.

**Data curation:** Sümeyye Belhan Çelik.

**Formal analysis:** Sümeyye Belhan Çelik, Esma Özkan.

**Investigation:** Sümeyye Belhan Çelik.

**Supervision:** Sümeyye Belhan Çelik, Esma Özkan.

**Visualization:** Sümeyye Belhan Çelik.

**Writing – original draft:** Sümeyye Belhan Çelik, Esma Özkan.
